# Potentiation of Acetylcholine-Induced Relaxation of Aorta in Male UC Davis Type 2 Diabetes Mellitus (UCD-T2DM) Rats: Sex-Specific Responses

**DOI:** 10.3389/fphys.2021.616317

**Published:** 2021-07-22

**Authors:** Farjana Akther, Md Rahatullah Razan, Sonali Shaligram, James L. Graham, Kimber L. Stanhope, Kaitlin N. Allen, José Pablo Vázquez-Medina, Peter J. Havel, Roshanak Rahimian

**Affiliations:** ^1^Department of Physiology & Pharmacology, Thomas J. Long School of Pharmacy, University of the Pacific, Stockton, CA, United States; ^2^Department of Molecular Biosciences, School of Veterinary Medicine, University of California, Davis, Davis, CA, United States; ^3^Department of Nutrition, University of California, Davis, Davis, CA, United States; ^4^Department of Integrative Biology, University of California, Berkeley, Berkeley, CA, United States

**Keywords:** sex differences, aorta, type-2 diabetes, nitric oxide, insulin resistance

## Abstract

Previous reports suggest that diabetes may differentially affect the vascular beds of females and males. The objectives of this study were to examine whether there were (1) sex differences in aortic function and (2) alterations in the relative contribution of endothelium-derived relaxing factors in modulating aortic reactivity in UC Davis Type 2 Diabetes Mellitus (UCD-T2DM) rats. Endothelium-dependent vasorelaxation (EDV) in response to acetylcholine (ACh) was measured in aortic rings before and after exposure to pharmacological inhibitors. Relaxation responses to sodium nitroprusside were assessed in endothelium-denuded rings. Moreover, contractile responses to phenylephrine (PE) were measured before and after incubation of aortic rings with a nitric oxide synthase (NOS) inhibitor in the presence of indomethacin. Metabolic parameters and expression of molecules associated with vascular and insulin signaling as well as reactive oxygen species generation were determined. Diabetes slightly but significantly impaired EDV in response to ACh in aortas from females but potentiated the relaxation response in males. The potentiation of EDV in diabetic male aortas was accompanied by a traces of nitric oxide (NO)- and prostanoid-independent relaxation and elevated aortic expression of small- and intermediate conductance Ca^2+^-activated K^+^ channels in this group. The smooth muscle sensitivity to NO was not altered, whereas the responsiveness to PE was significantly enhanced in aortas of diabetic groups in both sexes. Endothelium-derived NO during smooth muscle contraction, as assessed by the potentiation of the response to PE after NOS inhibition, was reduced in aortas of diabetic rats regardless of sex. Accordingly, decreases in pAkt and peNOS were observed in aortas from diabetic rats in both sexes compared with controls. Our data suggest that a decrease in insulin sensitivity *via* pAkt-peNOS-dependent signaling and an increase in oxidative stress may contribute to the elevated contractile responses observed in diabetic aortas in both sexes. This study demonstrates that aortic function in UCD-T2DM rats is altered in both sexes. Here, we provide the first evidence of sexual dimorphism in aortic relaxation in UCD-T2DM rats.

## Introduction

Over the past decade, obesity and type 2 diabetes (T2D) have reached epidemic levels worldwide, becoming one of the most challenging health problems in the 21st century (Tabish, 2007; Zheng et al., 2018). Cardiovascular diseases (CVDs) are one of the primary causes of morbidity and mortality in patients with diabetes (Brunner et al., 2005). Premenopausal women have a lower incidence of CVD when compared with age-matched men (Barrett-Connor, 1994). However, premenopausal women with diabetes not only lose the sex-based cardiovascular protection but also experience a higher relative risk of CVD compared to diabetic men (Steinberg et al., 2000). It has been established that hyperglycemia and diabetes affect female and male vascular beds differently. We previously reported sex differences in the development of vascular dysfunction in arteries of streptozotocin-induced type 1 diabetic rats (Zhang et al., 2012; Han et al., 2016). However, the pathophysiology of vascular dysfunction in T2D is likely to differ from that in type 1 diabetes.

With the increasing prevalence of T2D, creating effective preclinical models of the disease has become crucial for disease prevention and treatment. Dr. Havel and colleagues at the University of California (UC) Davis developed a validated rat model of T2D, the UC Davis Type 2 Diabetes Mellitus (UCD-T2DM) rats. UCD-T2DM rats exhibit all of the key features of the disease in humans such as polygenic adult-onset obesity, insulin resistance, intact leptin signaling, and spontaneous β-cell decompensation with preserved fertility in both sexes (Cummings et al., 2008; Kleinert et al., 2018).

Recently, we reported sex differences in the development of impaired vascular reactivity in mesenteric arteries from UCD-T2DM rats (Shaligram et al., 2020). Nevertheless, it remains to be established whether this reported sexual dimorphism is specific to the small arteries or is generalizable to larger conduit arteries in type 2 diabetic arteries. Thus, the initial aim of our study was to determine whether the aortic response to endothelium-dependent and independent vasodilators and vasoconstrictors varies with sex in UCD-T2DM rats.

Endothelium-dependent vasorelaxation (EDV) is considered a reproducible factor for assessing endothelial function (De Vriese et al., 2000). In diabetes, enhanced (Aloysius et al., 2012), impaired (Vanhoutte et al., 2009), and preserved EDV (Miller and Vanhoutte, 1991) have been reported. Altered EDV can result from alteration in synthesis or release of endothelium-derived relaxing factors (EDRF) [nitric oxide (NO), prostacyclin (PGI_2_), and endothelium-derived hyperpolarizing factor (EDHF)] or endothelium-derived contracting factors.

Nitric oxide has been considered a major contributor to EDV in large conduit arteries (Félétou, 2011), whereas EDHF plays a predominant role in small resistance arteries (Garland et al., 1995). In large conduit arteries, it is widely accepted that NO levels are reduced in diabetes (Han et al., 2014) and that changes in the level of endothelial NO synthase (eNOS) and/or increased generation of reactive oxygen species (ROS) such as superoxide may contribute to the reduction of NO production or bioavailability.

It has also been proposed that EDHF may play a role as a backup vasodilator in small resistance vessels when NO bioavailability is compromised (Brandes et al., 2000). The chemical identity of EDHF varies with vascular size, vascular bed and species (Leo et al., 2011). The classical EDHF pathway involves the opening of small- and intermediate-conductance calcium-activated potassium channels (SK_C__a_ and IK_Ca_) on the endothelium and the subsequent hyperpolarizing of smooth muscle cells *via* activation of Na-K-ATPase and/or K_ir_ channels or through myoendothelial gap junctions (Edwards et al., 1998; Parkington et al., 2002; Sandow et al., 2002). Previous studies have provided evidence of EDHF-type responses induced by acetylcholine (ACh) in rabbit conduit arteries that are potentiated by the elevation of cAMP but inhibited by disruption of gap junctions or a combination of SK_Ca_ and IK_Ca_ channel blockers (Griffith et al., 2002).

Overall, studies in various experimental models have evaluated the effects of diabetes on endothelial NO production. However, the sex-specific effects of T2D on the relative contribution of EDRF to the vascular reactivity of large conduit arteries remain unclear. Here, we examined changes in the relative importance of EDRF in modulating aortic relaxation in male and female UCD-T2DM rats.

Insulin resistance, a key element in the pathogenesis of T2D (Ormazabal et al., 2018), is associated with endothelial dysfunction by several mechanisms including oxidative stress. Here, we evaluated the responsiveness to insulin signaling by measuring the aortic expression of insulin receptor substrates (IRS-1 and IRS-2), total and phosphorylated levels of Akt, and eNOS. Since NADPH oxidases (NOX) are a potent cellular source of superoxide in the cardiovascular system (Drummond and Sobey, 2014), experiments were also carried out to determine the aortic expression of NOX subtypes. Moreover, ROS generation was determined in primary aortic endothelial cells isolated from male and female UCD-T2DM rats.

This study demonstrates that aortic function in UCD-T2DM rats is altered in both sexes. Here, we provide the first evidence of sexual dimorphism in aortic relaxation in UCD-T2DM rats.

## Materials and Methods

### Experimental Animals

Male and female UCD-T2DM rats were generated by breeding obese Sprague–Dawley (SD) rats with Zucker Diabetic Fatty (ZDF) lean rats that were homozygous wild type for the leptin receptor and had inherent β-cell defects. Rats were bred at the animal facility in the Department of Nutrition at the UC Davis (Cummings et al., 2008).

Rats were maintained with water and standard rodent chow food *ad libitum* at constant humidity and temperature, with a light/dark cycle of 12 h. After acclimation for 1 week at the animal facility at the University of the Pacific, animals were euthanized for experiments using carbon dioxide as the euthanasia agent, according to the recommendations from the 2013 AVMA Guidelines on Euthanasia and the NIH Guidelines for the Care and Use of Laboratory Animals: Eighth Edition (US National Institutes of Health, 2011). Age-matched male and female non-obese and non-diabetic SD rats (Simonsen Laboratories, Gilroy, CA, United States) (∼average 19–20 weeks old) were employed as controls for UCD-T2DM rats. Diabetic phase was determined by measuring blood glucose levels for three subsequent measurements using a standard glucose test meter (OneTouch, LifeScan, CA). Animals were considered diabetic when non-fasting blood glucose levels were higher than 300 mg/dl. The diabetic animals used in the study were diabetic for 35 ± 2.7 (males) and 31 ± 3.1 (females) days. UCD-T2DM rats exhibit insulin resistance prior to the onset of diabetes, similar to humans (Cummings et al., 2008).

All animal protocols were approved by the Animal Care Committee of the University of the Pacific and UC Davis Institutional Animal Care and Use Committee and complied with the Guide for the Care and Use of Laboratory Animals: Eighth Edition (US National Institutes of Health, 2011) and with ARRIVE guidelines.

### Measurement of Metabolic Parameters in the Plasma

Blood glucose levels were measured in 12-h fasted rats using a standard glucose test meter (OneTouch, LifeScan, CA) and triglycerides were measured by using an Accutrend Plus System (hand-held point-of-care device) and specific test strips (Roche Farma, Barcelona, Spain) with a drop of blood collected from the tail vein. Blood samples were collected from intracardiac puncture and obtained in tubes containing heparin as an anticoagulant. Plasma was obtained by centrifugation at 10,000 × *g* for 5 min at 4°C and stored at −80°C until used. Insulin levels were determined in plasma samples by using ELISA kits according to the manufacturer’s protocol (Spi Bio, Montigny Le Bretonneux, France). Insulin sensitivity index (ISI) was determined from fasting plasma glucose and insulin using the following formula: ISI = [2/(blood insulin (nM) × blood glucose (μM) + 1)] (Sangüesa et al., 2017). HbA1c was measured using the Bayer A1cNow test kit, according to the manufacturer’s instructions, and animals with A1c levels greater than 6.5% on two separate tests were considered diabetic.

### Measurement of Arterial Tension

The thoracic aorta was cut into 2-mm rings after being excised and cleaned off adhering connective tissues. To measure isometric tension, the rings were suspended horizontally between two stainless steel hooks in individual organ baths containing 20 ml of Krebs buffer (in mM: 119 NaCl, 4.7 KCl, 1.18 KH_2_PO_4_, 1.17 MgSO_4_, 24.9 NaHCO_3_, 0.023 EDTA, 1.6 CaCl_2_, and 6.0 glucose) at 37°C bubbled with 95% O_2_ and 5% CO_2_. Isometric tension was continuously monitored with a computer-based data acquisition system (PowerLab; ADInstruments, Colorado Springs, CO, United States). To develop a stable basal tone, aortic rings were equilibrated under 1*g* resting tension for 40 min. Rings were stimulated two times with 80 mM KCl every 20 min until maximum contraction was achieved. The ability of ACh (10 μM) to induce relaxation of phenylephrine (PE, 2 μM) pre-contracted vessels was taken as evidence for the preservation of an intact endothelium. For the relaxation studies, an equal submaximal concentration of PE (2 μM) was used in both males and females.

### Relaxation Responses to ACh

Aortic rings were precontracted with PE (2 μM), which represented a concentration that produced 80% of the maximal effect (EC_80_). The concentration response curves (CRCs) were obtained by the addition of increasing concentrations of ACh (10^–8^ to 10^–5^ M). In addition, CRCs to ACh were obtained before and after 20 min incubation with indomethacin (Indo; 10 μM; dissolved in DMSO), a cyclooxygenase (COX) inhibitor, Indo plus 1H-[1,2,4] oxadiazolo [4,3-a] quinoxalin-1-one (ODQ; 10 μM), an inhibitor of soluble guanylate cyclase (sGC), and finally after incubation with Indo, ODQ, and *N*-nitro-L-arginine (L-NNA; 100 μM), a non-selective NO synthase (NOS) inhibitor. Tissues were washed with Krebs buffer between each CRC to allow the rings to return to basal tone.

### Relaxation Responses to Sodium Nitroprusside (SNP)

CRCs to SNP (10^–9^ to 10^–5^ M), a NO-donor, were obtained in endothelium-denuded aortic rings pre-contracted with PE (2 μM) taken from all experimental groups.

### Contractile Responses to PE

The constrictor CRCs to PE (10^–8^ to 10^–5^ M) were generated before and after incubation with N^ω^ -nitro-L-arginine methyl ester (L-NAME, 200 μM), a NOS inhibitor, in the presence of Indo (10 μM, dissolved in DMSO), a COX inhibitor. Tissues were washed with Krebs buffer between each CRC to allow the rings to return to basal tone. A vehicle-only (no drugs present) study was performed simultaneously in aortic rings from the same animal (data not shown).

### Western Blot Analysis

Aortic tissue samples were micronized through freezing with liquid nitrogen and grinding with a mortar and pestle as previously described (Baena et al., 2015). To obtain total protein extract, samples were incubated with RIPA buffer (Sigma-Aldrich, St. Louis, MO, United States) containing Protease Inhibitor Cocktail (UltraCruz, Santa Cruz Biotechnology, Dallas, TX, United States) for 1.5 h at 4°C and centrifuged at 15,000 × *g* for 15 min at 4°C, and supernatants were collected. Protein concentrations were determined by the bicinchoninic acid (BCA) assay.

Protein (20–30 μg) was subjected to sodium dodecyl sulfate polyacrylamide gel electrophoresis (SDS-PAGE). Proteins were then transferred to 0.45-μm nitrocellulose membranes (Bio Rad Laboratories Inc., Hercules, CA, United States), blocked for 1 h at room temperature with 5% w/v BSA in 0.1% Tween-20 Tris-buffered saline (TBS), and incubated overnight at 4°C with primary antibodies. All primary antibodies were diluted 1:1000 unless otherwise noted. Primary antibodies for endothelial NO synthase (eNOS), phospho-eNOS (p-eNOS) (Ser-1177), V-akt murine thymoma viral oncogene homolog-2 (Akt), phospho-AKT(p-AKT) (Ser-473), and insulin receptor substrates 1 and 2 (IRS-1 and IRS-2) were supplied by Cell Signaling (Boston, MA, United States). Antibodies against NOX1, NOX4, KCNN3 (SK_Ca_), and KCNN4 (IK_Ca_) were obtained from Abcam (Cambridge, MA, United States). Incubation with secondary antibody (LI-COR donkey anti-Rabbit IgG IRDye 680 or anti-mouse IgG IRDye 800CW, 1:10,000) was performed in the blocking buffer for 1 h at room temperature. Before analyzing, the membrane was washed four times with TBS containing 0.1% Tween-20. Detection was done by using a LI-COR Odyssey imaging system (Lincoln, NE, United States). The bands were quantified by densitometry using Image Studio Lite software. To confirm the uniformity of protein loading, blots were incubated with GAPDH and β-actin antibodies (Cell Signaling, Boston, MA, United States) and were normalized for GAPDH and β-actin (data expressed as fold change from control group).

### Measurement of ROS Generation by Rat Aortic Endothelial Cells

#### Primary Cell Isolation and Culture

Aortas were cut open lengthwise to expose the endothelial surface. Vessels were incubated in a collagenase II (Worthington) solution (2 mg/ml in DMEM) at 37°C with the endothelial surface facing down for 30 min. Collagenase was blocked 1:1 with complete endothelial cell growth medium [DMEM supplemented with 10% fetal bovine serum, 1% Antibiotic-Antimycotic solution (Gibco, MA), 4 μg/ml endothelial cell growth supplement (Corning, 354006), 1% Non-essential amino acids (Gibco, MA), and 10 mM HEPES (Gibco, MA)]. The endothelial surface of each vessel was scraped into fibronectin-coated tissue culture dishes containing complete medium. Cultures were expanded and frozen at passage 1. The endothelial phenotype of the preparation was confirmed by evaluating cellular uptake of the endothelium-specific marker DiI-acetylated low-density lipoprotein. Experiments were conducted in cells obtained from three control and three diabetic male and four control and four diabetic female rats. The day before the experiments, cells were plated in commercial endothelial cell growth medium (ScienCell, CA) supplemented with 25 mM glucose (ScienCell, CA).

#### Assays for Oxidant Generation in Intact Cells

H_2_O_2_ generation was measured using Amplex Red and horseradish peroxidase (HRP) as previously described (Vázquez-Medina et al., 2016). Briefly, cells were incubated with 50 μM Amplex Red (Thermo Fisher, MA) and 2.5 U/ml HRP (Sigma) for 30 min at 37°C. The medium was collected, and absorbance was measured at 572 nm. At the end of the experiments, the cells were dissociated from the dishes. Protein content was measured by the BCA gold assay (Thermo Scientific, MA) and results were normalized to protein content. Intracellular oxidant generation was monitored by fluorescence microscopy using CellROX reagent (Thermo Scientific, MA). Cells loaded with 5 μM CellROX and NucBlue (Thermo Fisher, MA) were incubated for 30 min at 37°C. Cells were rinsed three times and imaged using an inverted fluorescence microscope (Zeiss Axio Observer) fitted with a 20× objective and Zen software. Fluorescence intensity in five fields per sample was quantified using ImageJ (NIH) and normalized to cell number.

### Statistical Analysis

All values are expressed as mean ± standard error of the mean (SEM). Relaxation responses to each concentration of ACh and SNP were calculated as the percentage of relaxation from maximum PE contraction. Similarly, the recorded increase in the force of contraction was calculated as the percentage of maximum contraction obtained with PE at the highest dose or as changes in tension with increasing concentration of PE in the aortic rings. The concentration of agonist that produced half of the maximum effect (*E*_max_) was expressed as EC_50_ and calculated by a sigmoidal dose–response model (for variable slope) using GraphPad Prism v7 (GraphPad Software Inc., San Diego, CA, United States). Sensitivity to each agonist was expressed as pD_2_ values (−log [EC_50_]), which were normally distributed. The area under the curve (AUC) was determined using GraphPad Prism 8 with trapezoidal technique. To compare the effect of different EDRFs such as COX, the ACh results were expressed as differences in the area under the concentration response curve (ΔAUC) between control (absence of Indo) and experimental (presence of Indo) conditions. One-way ANOVA was used to compare means among experimental groups (e.g., EC_50_, *E*_max_, and metabolic data). When the one-way ANOVA test returned *p* < 0.05, *post hoc* analyses were performed using Tukey’s test. Comparison of CRCs between two groups was done using two-way ANOVA, with one factor being concentration and the other being group (male vs. female and control vs. diabetic). When the ANOVA test returned *p* < 0.05, *post hoc* analyses were performed using Bonferroni’s or Tukey’s test. Comparison of CRCs in a pre/post-test format within a group was done using two-way ANOVA with repeated measures. Three-way ANOVA with factors being sex, diabetes, and drugs (male vs. female, diabetic vs. non-diabetic and no drug vs. drug treatment) was used to compare group means. When the ANOVA test returned *p* < 0.05, *post hoc* analyses were performed using Tukey’s test.

For ROS generation assays, means were compared between control and diabetic groups using unpaired Student’s *t*-tests for both males and females. Normality was confirmed using the Shapiro–Wilk test. Statistical analyses were conducted using GraphPad Prism v8.4.3. Values were considered different when *p* < 0.05. Student’s unpaired *t*-test was used for comparisons of two group means (e.g., protein expression studies).

## Results

### Metabolic Parameters and Insulin Signaling in UCD-T2DM Rats

Body weights of both male and female diabetic rats were significantly higher compared with the respective non-diabetic controls (Table 1). Accordingly, the weight of intra-abdominal adipose tissue, as well as its ratio to body weight (adiposity), was higher in diabetic rats than in non-diabetic control groups for both sexes. Moreover, male and female UCD-T2DM rats had higher triglyceride levels in plasma than did the respective non-diabetic controls. When compared to male UCD-T2DM rats, female UCD-T2DM rats had significantly higher circulating triglyceride levels and adiposity. Furthermore, both fasting glucose and HbA1c levels were higher in male and female diabetic rats compared to their respective non-diabetic controls. Fasting plasma insulin concentration was significantly higher in female diabetic rats compared with those in both the non-diabetic female control and male diabetic groups (Table 1). Similar to the previous report (Shaligram et al., 2020), there was no difference in plasma insulin levels in male diabetic rats when compared with their respective non-diabetic controls. However, the ISI was significantly lower in diabetic groups, regardless of sex, indicating that insulin signaling may be impaired in diabetic groups of both sexes. When compared to male UCD-T2DM rats, female UCD-T2DM rats had a lower ISI (Table 1).

**TABLE 1:** Body weight and adipose weight, blood glucose levels, HbA1c, and other metabolic parameters of male and female control and diabetic rats.

	*n*	Male control	Male diabetic	Female control	Female diabetic
Body weight (g)	9–12	314.40 ± 21.7	529.60 ± 20.8^∗^	210.47 ± 6.4^#^	403.99 ± 6.8^∗^
Adipose tissue (g)	9–12	1.25 ± 0.1	11.77 ± 0.9^∗^	1.56 ± 0.2	14.06 ± 1.8^*#^
Adipose tissue/body weight (g)	9–12	0.0043 ± 0.0	0.02 ± 0.0^∗^	0.007 ± 0.0	0.036 ± 0.0^*#^
Triglyceride (mmol/L)	9–12	1.30 ± 0.1	1.94 ± 0.2^∗^	1.27 ± 0.0	3.62 ± 0.4^*#^
Blood glucose (mg/dl)	9–12	132.25 ± 8.4	302.23 ± 29.3^∗^	144.88 ± 12.5	388.73 ± 34.3^∗^
HbA1c level	9–12	4.23 ± 0.1	10.95 ± 0.8^∗^	4.43 ± 0.0	9.99 ± 0.9^∗^
Insulin (ng/ml)	8–10	0.91 ± 0.5	0.96 ± 0.2	0.49 ± 0.1	5.71 ± 1.9^*#^
ISI	8–10	1.35 ± 0.1	0.60 ± 0.1^∗^	0.89 ± 0.2	0.10 ± 0.0^*#^

*Data are expressed as mean ± SEM.*

n, number of rats per group.

*p < 0.05 (vs. control, same sex), ^#^*p* < 0.05 (vs. male, respective group), using one-way ANOVA followed by Tukey’s *post hoc* test.

*Insulin sensitivity index (ISI) = [2/(blood insulin (nM) × blood glucose (μM) + 1]. HbA1c = glycated hemoglobin (A1c).*

**FIGURE 1:**
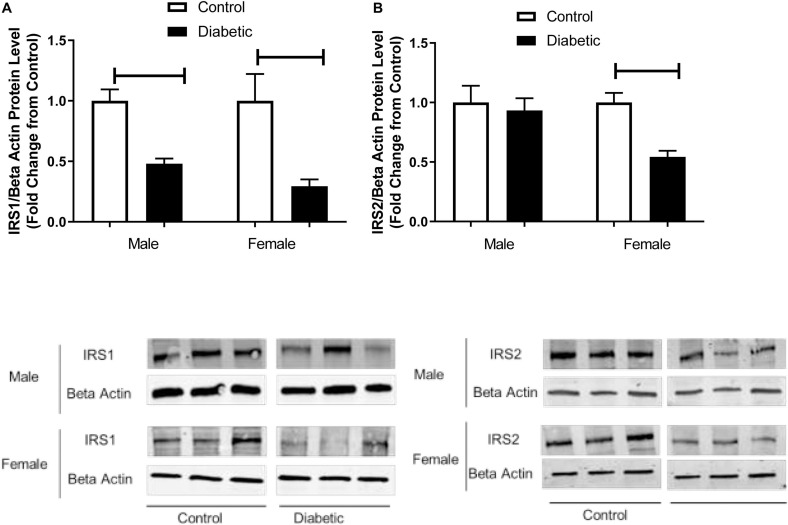
Western blot analysis of IRS1 and IRS2 expression in control and UCD-T2DM rat aorta. Protein levels of **(A)** aortic insulin receptor substrate-I (IRS1) and **(B)** insulin receptor substrate-2 (IRS2) from the samples of male and female control and diabetic rats. IRS1 **(A)** and IRS2 **(B)** were quantified by densitometry and normalized to corresponding beta actin. Values are represented as mean ± SEM. Each bar represents the values obtained from n = 4–5 animal s per group. To show representative bands, images from different parts of the same gel have been juxtaposed, indicated by white dividing lines. Capped lines indicate significant differences between two groups (*p* < 0.05), as analyzed by unpaired Student’s *t*-test.

The reduced ISI observed in the current study prompted us to analyze the expression of the main insulin signal transducers, insulin receptor substrate-1 (IRS-1) and insulin receptor substrate-2 (IRS-2), in aortic tissue. As shown in Figure 1A, IRS-1 expression was significantly reduced in both male and female diabetic groups (by 0.5-fold and 0.6-fold in male and female diabetic groups, respectively). In contrast, only the female diabetic group displayed reduced IRS-2 protein expression (0.5-fold) compared to the sex-specific non-diabetic control (Figure 1B).

### Relaxation Responses to ACh in UCD-T2DM Rat Aortas

A sex difference was observed in aortic relaxation responses to ACh in non-diabetic control rats. Both sensitivity, as assessed by −log [EC_50_] (pD_2_) value, and *E*_max_ to ACh were significantly higher in female than in male aortas (Table 2). In controls, the pD_2_ to ACh was 6.57 ± 0.1 in male and 7.20 ± 0.1 in female aortas; the ACh *E*_max_ was 81.14% ± 1.6% in male and 94.72% ± 1.9% in female aortas (*n* = 6–12 per group, *p* < 0.05, one-way ANOVA).

**TABLE 2:** pD_2_ and *E*_max_ to acetylcholine (ACh) in aortic rings from male and female control and diabetic rats.

Ach	*n*	pD_2_	*E*_max_ (%)
Male control	12	6.57 ± 0.1	81.14 ± 1.6
Male diabetic	11	7.14 ± 0.0*	94.04 ± 0.9*
Female control	6	7.20 ± 0.1^#^	94.72 ± 1.9^#^
Female diabetic	7	7.07 ± 0.1	85.92 ± 2.0^*#^

*Data are expressed as mean ± SEM.*

n, number of rats per group.

*p < 0.05 (vs. control, same sex), ^#^*p* < 0.05 (vs. male, respective group), analyzed using one-way ANOVA followed by Tukey’s *post hoc* test.

**FIGURE 2:**
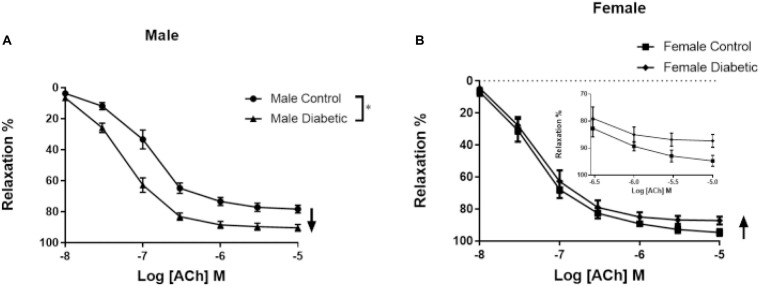
Concentration-response curves to acetylcholine (ACh) in control and UCD-T2DM rat aorta. Relaxation responses to cumulative concentrations of ACh (10^–8^ to 10^–5^ M) in intact aortic rings pre-contracted with phenylephrine (PE, 2 μM) from male **(A)** and female **(B)** control and diabetic rats. Data are expressed as mean ± SEM. n = 6–12 animals per group. *p < 0.05 between two groups analyzed using two-way ANOVA followed by Bonferroni’s post hoc test.

When compared to the male non-diabetic control group, a potentiated relaxation response to ACh was observed in the male diabetic group (Figure 2A). Both the pD_2_ and *E*_max_ of aortic rings to ACh were significantly enhanced in the male diabetic group (Table 2). The pD_2_ to ACh was 6.57 ± 0.1 in control males and 7.14 ± 0.0 in diabetic males; the ACh *E*_max_ was 81.14% ± 1.6% in control males and 94.04% ± 0.9% in diabetic males (*n* = 11–12 per group, *p* < 0.05, one-way ANOVA). However, the *E*_max_ but not the sensitivity of aortic rings to ACh was reduced slightly but significantly in the female diabetic group compared to its respective control and the diabetic male group (Figure 2B and Table 2). The ACh *E*_max_ was 94.7% ± 1.9% in control females and 85.9% ± 2.0% in diabetic females (*n* = 6–7 per group, *p* < 0.05, one-way ANOVA).

### Relative Contribution of EDRF to ACh-Induced Relaxation in UCD-T2DM Rat Aorta

The relative contributions of PGI_2_, cGMP, and NO to vasorelaxation induced by ACh were estimated by sequentially inhibiting COX, sGC, and NOS. Specifically, EDV to ACh (10^–8^ to 10^–5^ M) in rat aortic rings pre-contracted with PE (2 μM) was obtained before and after pretreatment with Indo (10 μM), followed by the addition of ODQ (10 μM) and L-NNA (100 μM). When ODQ was added, the EDV reduction is thought to represent the impact of NO-dependent cGMP on EDV (Pieper and Siebeneich, 1997). Finally, addition of L-NNA, after inhibition of sGC by ODQ, represents the impact of NO independent of cGMP (Bolotina et al., 1994), and the slight remaining EDV to ACh is referred to as the L-NNA, Indo-insensitive component-type relaxation (Feletou and Vanhoutte, 1988).

**FIGURE 3:**
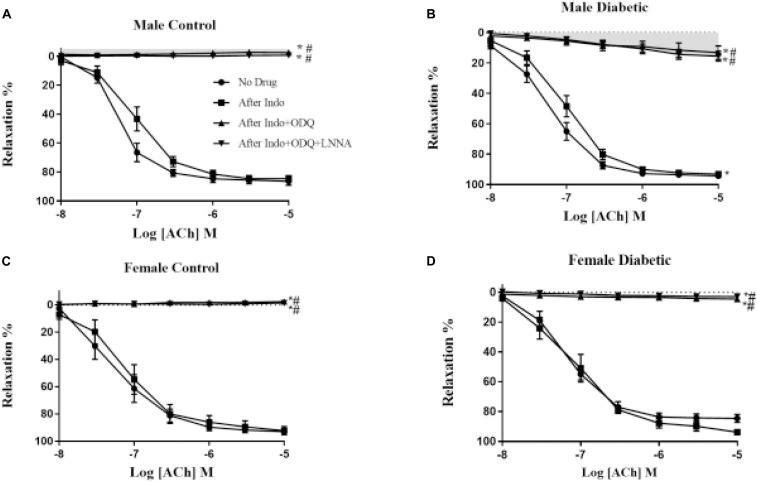
Endothelium-derived relaxing factors (EDRF) contribution to acetylcholine (ACh)− induced relaxation responses in control and UCD-T2DM rat aorta. Effects of inhibiting cyclooxygenase, soluble guanylyl cyclase and nitric oxide synthase on ACh-induced vasorelaxation in aortic rings taken from **(A)** male control and **(B)** male diabetic, **(C)** female control and **(D)** female diabetic rats. ACh relaxation was measured in the presence of indomethacin (Indo, 10 μM), followed by addition of ODQ (10 μM), and then with N-nitro-L-arginine (L-NNA; 100 μM). Data are expressed as mean ± SEM. *p < 0.05 vs. no drug; ^#^P < 0.05 vs. Indo; analyzed using two-way ANOVA with repeated measures followed by Bonferroni post hoc test (n = 5–8 per group). Light gray shaded area: contribution of endothelium-derived hyperpolarizing factor (EDHF)-type to endothelium-dependent vasorelaxation (EDV).

The administration of Indo to block COX activity had no apparent effects on pD_2_ and *E*_max_ to ACh, regardless of sex or diabetes status. The ΔAUC, defined as the difference in the AUC between the CRC to ACh before and after Indo, was not different between UCD-T2DM groups and respective non-diabetic control groups in either sex (Figures 3A–D and Table 3). Addition of ODQ completely blocked the remaining relaxation in all experimental groups except for the male diabetic group (Figure 3B). After adding ODQ, a slight but significant relaxation response remained in aortic rings of the male diabetic group compared to the control group (Figure 3B vs. Figure 3A). The *E*_max_ to ACh in male control and male diabetic aortas was 2.22% ± 0.2% and 10.14% ± 0.5%, respectively (*p* < 0.05, one-way ANOVA) (Table 3, third column). To examine whether the slight residual ACh-induced relaxation in male diabetic aortas may be due to the direct action of NO (independent of cGMP), a NOS inhibitor was used. The addition of L-NNA, a non-selective NOS inhibitor, had no apparent effect on the remaining Indo- and ODQ-resistant relaxation in male diabetic aortas (Figure 3B). After the addition of L-NNA, there was still a significant difference in the *E*_max_ to ACh between male control and male diabetic groups. The *E*_max_ to ACh was 0.43% ± 0.4% in male control and 13.38% ± 1.2% in male diabetic rats (*p* < 0.05, one-way ANOVA) (Table 3, fourth column). The remaining AUC after addition of L-NNA in the male diabetic group was significantly different from the male control group, suggesting a slight role of NO-PGI_2_-independent relaxation responses in this group (Table 3, fourth column, Figure 3B, gray shaded area).

**TABLE 3:** Area under the curve (ΔAUC), Sensitivity (pD_2_: -logEC50) and maximum response (*E*_max_) to ACh in rat aortic rings from male and female control and diabetic rats.

Groups	No drug			Indo			Indo + ODQ			Indo + ODQ + L-NNA		
	pD_2_	*E*_max_ (%)	ΔAUC	pD_2_	*E*_max_ (%)	ΔAUC	pD_2_	*E*_max_ (%)	ΔAUC	pD_2_	*E*_max%_	AUC
Male control	7.04 ± 0.1	83.82 ± 2.9	ND	6.72 ± 0.0	81.90 ± 2.31	28.2 ± 4.7	ND	2.22 ± 0.2^ab^	163.99 ± 9.2	ND	0.43 ± 0.4^ab^	2.79 ± 1.2
Male diabetic	7.18 ± 0.0	94.16 ± 1.1*	ND	6.95 ± 0.1	93.97 ± 0.3	23.7 ± 0.9	ND	10.14 ± 0.5*^ab^	166.05 ± 5.1	ND	13.38 ± 1.2*^ab^	11.15 ± 1.6*
Female control	7.31 ± 0.0	92.50 ± 0.7^#^	ND	7.29 ± 0.0	92.16 ± 2.8	6.35 ± 1.0	ND	2.38 ± 1.1^ab^	185.60 ± 15.8	ND	1.51 ± 0.6^ab^	3.28 ± 1.1
Female diabetic	7.19 ± 0.0	84.50 ± 2.7*^#^	ND	7.20 ± 0.0	93.78 ± 0.9	9.71 ± 4.6	ND	4.39 ± 0.6^ab#^	187.59 ± 13.5	ND	2.72 ± 0.7^ab#^	3.21 ± 1.2^#^

A comparison of the ΔAUC, sensitivity (pD_2_), and maximum response (*E*_*max*_) to acetylcholine in the absence (no drug) or in the presence of Indo, Indo + ODQ, and Indo + ODQ + L-NNA in aortic rings from male and female control and diabetic rats. Data are expressed as mean ± SEM.

*p < 0.05 (vs. control, same sex), ^#^*p* < 0.05 (vs. male in respective group) (one-way ANOVA followed by Tukey’s *post hoc* test); ^*a*^*p* < 0.05 vs. no drug control within each group, ^*b*^*p* < 0.05 vs. indo within each group (two-way ANOVA with repeated measures followed by Tukey’s *post hoc* test), *n* = 5–8 per group.

*ND, not determined.*

**FIGURE 4:**
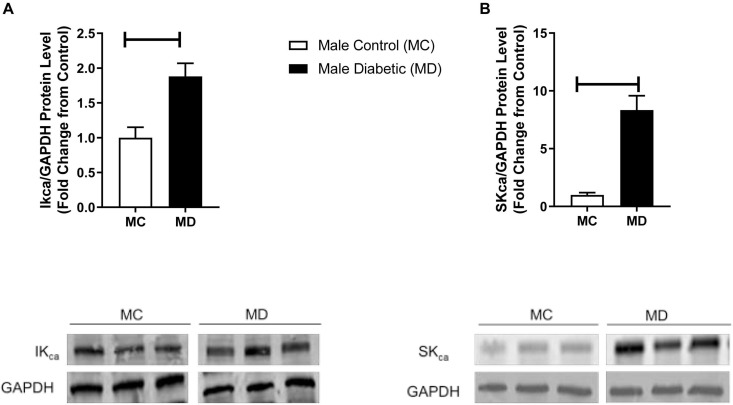
Western blot analysis IK_Ca_ and SK_Ca_ expression in control and UCD-T2DM rat aorta. Protein levels of **(A)** aortic intermediate conductance calcium activated potassium channel (IK_Ca_) and **(B)** small conductance calcium activated potassium channels (SK_Ca_) were measured from the samples of male control and diabetic rats. IK_Ca_ **(A)** and SK_Ca_ **(B)** were quantified by densitometry and normalized to corresponding GAPDH. Values are represented as mean ± SEM. Each bar represents the values obtained from *n* = 4–5 per group. To show representative bands, images from different parts of the same gel have been juxtaposed, indicated by white dividing lines. Capped lines indicate significant differences between two groups (*p* < 0.05), as analyzed by unpaired Student’s *t*-test. MC, male control; MD, male diabetic.

It is well known now that smooth muscle hyperpolarization results indirectly from the opening of endothelial SK_Ca_ and IK_Ca_ channels (McNeish et al., 2006). Therefore, an elevated NO-PGI_2_-independent-type relaxation in aortas from the male diabetic group could be expected to result from significant overexpression of these hyperpolarizing K_Ca_ channels on the endothelium (Gillham et al., 2007). Next, Western blot analysis revealed that the expression of both SK_Ca_ and IK_Ca_ was significantly upregulated (by 9.0-fold and by 1.0-fold, respectively) in the aortic tissues from male diabetic rats compared with those in non-diabetic controls (Figures 4A,B).

### Relaxation Responses to SNP in UCD-T2DM Rat Aorta

The smooth muscle sensitivity to NO was investigated by generating CRC to SNP (10^–9^ to 10^–5^ M) in endothelium-denuded aortic rings. No significant changes in either pD_2_ values or *E*_max_ of SNP were observed in diabetic animals of either sex. The pD_2_ values to SNP was 8.06 ± 0.0 in male control, 8.19 ± 0.1 in male diabetic, 8.50 ± 0.0 in female control, and 8.32 ± 0.0 in female diabetic animals. The *E*_max_ to SNP in male control and male diabetic animals was 100.15% ± 0.3% and 101.46% ± 1.4%, respectively. The *E*_max_ to SNP in female control and female diabetic animals was 104.34% ± 3.2% and 100.18% ± 0.4%, respectively.

### Contractile Responses to PE in UCD-T2DM Rat Aorta

**FIGURE 5:**
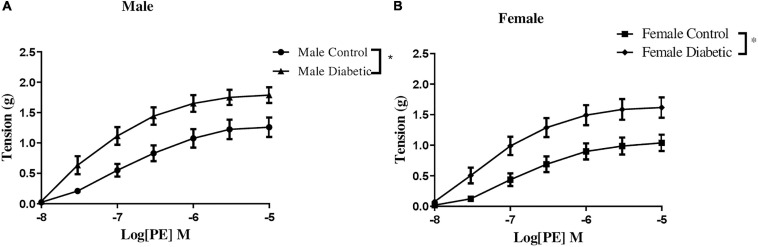
Concentration-response curves to phenylephrine (PE) in control and UCD-T2DM rat aorta. Contractile responses to cumulative concentrations of PE (10^–8^ to 10^–5^ M) in intact aortic rings of **(A)** male and **(B)** female control and diabetic rats. Data are expressed as mean ± SEM. *n* = 5–6 per group. *p < 0.05 between two groups analyzed using two-way ANOVA followed by Bonferroni’s *post hoc* test.

Contractile responses to an α-adrenoceptor agonist (PE) were analyzed by measuring the CRC to PE (10^–8^ to 10^–5^ M). There were no sex differences in PE contractile responses in aortic rings from non-diabetic control groups (Figure 5). However, both maximal tension developed in response to PE (Tension_max_) and the sensitivity to PE in aortic rings were significantly enhanced in aortic rings of diabetic groups compared with the non-diabetic control rats, regardless of sex (Figures 5A,B and Table 4).

**TABLE 4:** pD_2_ and Tension_max_ to phenylephrine (PE) in aortic rings from male and female control and diabetic rats.

PE	*n*	pD_2_	Tension_max_ (g)
Male control	5	6.81 ± 0.0	1.26 ± 0.1
Male diabetic	5	7.19 ± 0.0*	1.79 ± 0.1*
Female control	5	6.78 ± 0.0	1.04 ± 0.1
Female diabetic	6	7.14 ± 0.0*	1.53 ± 0.1*

*Data are expressed as mean ± SEM.*

n, number of rats per group.

*p < 0.05 (vs. control, same sex), analyzed using one-way ANOVA followed by Tukey’s *post hoc* test.

Next, the CRC to PE (10^–8^ to 10^–5^ M) was determined in aortic rings before and after pretreatment with the NO synthase inhibitor, L-NAME (200 μM), in the presence of Indo (10 μM). The changes in the contractile response to PE after the addition of L-NAME reveal the role of endothelium-derived NO during smooth muscle contraction in response to PE, as previously reported by us (Zhang et al., 2012; Sangüesa et al., 2017) and others (Hayashi et al., 1992; Dora et al., 2000).

**FIGURE 6:**
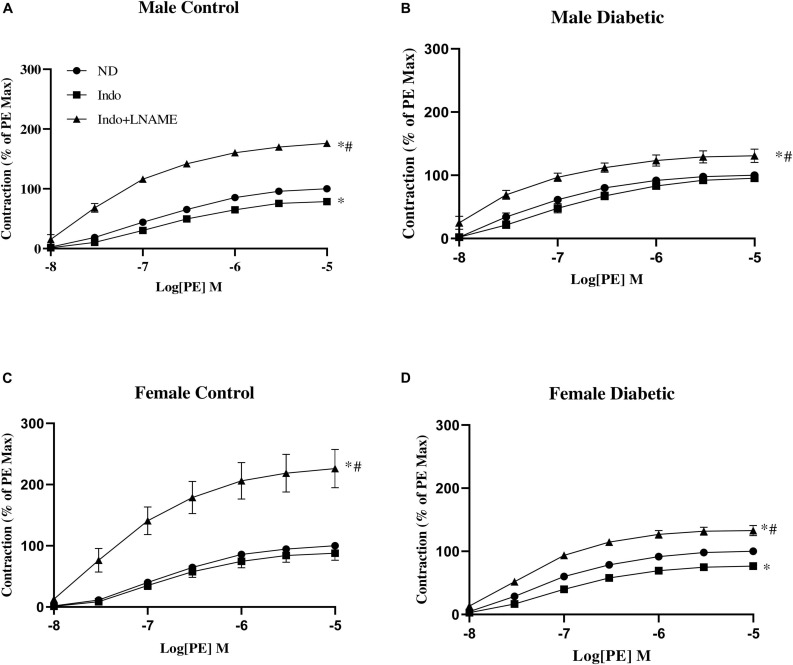
Concentration-response curves to phenylephrine (PE) in intact aortic rings from **(A)** male control, **(B)** male diabetic, **(C)** female control, and **(D)** female diabetic rats. Contraction to PE was measured in absence of any drug (ND) or in presence of indomethacin (Indo, 10 μM) followed by addition of *N*-Nitro-L-arginine methyl ester (Indo + L-NAME, 200 μM). Results are expressed as a percent of the maximal response to PE (10 μM) obtained in the absence of any drug. Data are expressed as mean ± SEM, analyzed using two-way ANOVA with repeated measures followed by Tukey’s *post hoc* test: ^∗^*p* < 0.05 vs. ND; ^#^*p* < 0.05 vs. Indo, *n* = 6–8 per group.

The administration of Indo to block COX metabolites slightly but significantly reduced *E*_max_ to PE in aortas from male control and female diabetic groups, suggesting a slight elevation of the contractile metabolite of COX in this group, with no apparent effect on the maximal contractile response in female control and male diabetic groups (Figure 6 and Table 5). The addition of L-NAME resulted in a significant increase of the contractile responses to PE in all experimental groups (Figure 6). However, ΔAUC (the difference in AUC between PE CRC before and after L-NAME) was lower in aortas of the diabetic rats compared with the control group, regardless of sex (Table 5).

**TABLE 5:** *E*_max_, Tension_max_, pD_2_, and ΔAUC to phenylephrine (PE) in aortic rings from male and female control and diabetic rats.

	*E*_*max (%)*_	Tension_max_ (g)	pD_2_	ΔAUC
**Male control (*n* = 6)**				
ND	100	1.26 ± 0.1	6.81 ± 0.0	
Indo	78.72 ± 4.8^a^	1.09 ± 0.1	6.74 ± 0.0	239.10 ± 5.0
Indo + L-NAME	176.15 ± 3.6^b^	2.47 ± 0.2^b^	7.29 ± 0.0^b^	
**Male diabetic (*n* = 8)**				
ND	100	1.79 ± 0.1^c^	7.19 ± 0.0^c^	
Indo	95.39 ± 5.5	1.66 ± 0.1	6.95 ± 0.1	103.66 ± 18.1^∗^
Indo + L-NAME	132.58 ± 8.7^bc^	2.26 ± 0.1^b^	7.46 ± 0.0^b^	
**Female control (*n* = 6)**				
ND	100	1.04 ± 0.1	6.78 ± 0.0	
Indo	87.83 ± 11.6	0.93 ± 0.1	6.76 ± 0.0	319.44 ± 59.8
Indo + L-NAME	226.19 ± 31.2^b^	2.25 ± 0.2^b^	7.21 ± 0.1^b^	
**Female diabetic (*n* = 7)**				
ND	100	1.53 ± 0.1^c^	7.14 ± 0.0^c^	
Indo	78.94 ± 2.2^a^	1.19 ± 0.1	7.01 ± 0.0	167.42 ± 6.8*
Indo + L-NAME	134.23 ± 9.4^bc^	1.98 ± 0.1^b^	7.35 ± 0.0^b^	

*Data are expressed as mean ± SEM. Three-way ANOVA with factors being sex, diabetes, and drugs were used to compare among group means of *E*_*max*_ (%), Tension_*max*,_ and pD_2_.*

*^*a*^*p* < 0.05 vs. (ND), ^*b*^*p* < 0.05 vs. (Indo); ^*c*^*p* < 0.05 (vs. control, same sex); analyzed using three-way ANOVA followed by Tukey’s *post hoc* test.*

ΔAUC, differences of area under the concentration–response curve with or without L-NAME in the presence of indo.

*p < 0.05 (vs. control, same sex), analyzed using two-way ANOVA followed by Tukey’s *post hoc* test. *n* = 6–8 per group.

### Intracellular Pathways Related to Vascular Function in UCD-T2DM Rat Aortas

**FIGURE 7:**
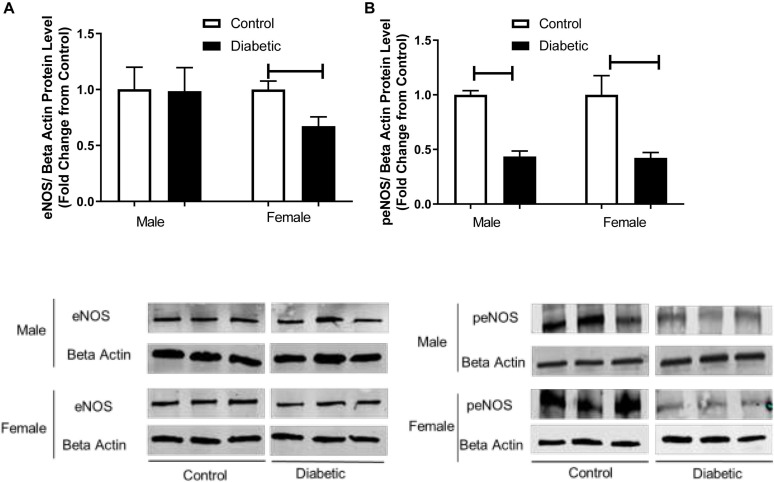
Western blot analysis of eNOS and peNOS expression in control and UCD-T2DM rat aorta. Protein levels of **(A)** total endothelial nitric oxide synthase (eNOS) and **(B)** phosphorylated eNOS (peNOS) in aortic samples from male and female control and diabetic rats. eNOS **(A)** and peNOS **(B)** were quantified by densitometry and normalized to corresponding beta actin. Each bar represents the mean ± SEM of values obtained from *n* = 4–5 animals per group. To show representative bands, images from different parts of the same gel have been juxtaposed, indicated by white dividing lines. Capped lines indicate significant differences between two groups (*p* < 0.05), as analyzed by unpaired Student’s *t*-test.

To investigate the mechanism by which endothelium-derived NO generation in response to PE might be reduced in diabetic animals, eNOS activation by phosphorylation was investigated by Western blot analysis. As shown in Figure 7B, the phosphorylation of eNOS at Ser-1177 was significantly reduced in aortic tissue from diabetic rats relative to controls, regardless of sex. Although the expression of total eNOS showed no significant difference between diabetic males and controls, its levels were significantly reduced in aortas from diabetic females (Figure 7A).

**FIGURE 8:**
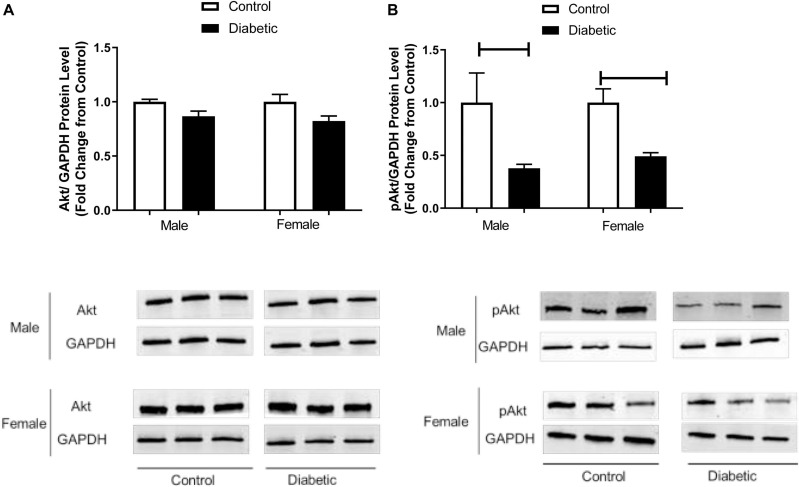
Western blot analysis of Akt and pAkt expression in control and UCD-T2DM rat aorta. Protein levels of aortic **(A)** total V-akt murine thymoma viral oncogene homolog-2 (Akt) and **(B)** phosphorylated V-akt murine thymoma viral oncogene homolog-2 (pAkt) from the samples of male and female control and diabetic rats. Akt **(A)** and pAkt **(B)** were quantified by densitometry and normalized to corresponding GAPDH. Each bar represents the mean ± SEM of values obtained from *n* = 4–5 animals per group. To show representative bands, images from different parts of the same gel have been juxtaposed, indicated by white dividing lines. Capped lines indicate significant differences between two groups (*p* < 0.05), as analyzed by unpaired Student’s *t*-test.

Vascular dysfunction could also be related to insulin resistance, and our results suggest that insulin signaling could be impaired in diabetic rats in both sexes. Next, we determined the expression of pAkt, which is downstream of IRS and an upstream mediator of eNOS phosphorylation at Ser-1177 in aortic tissues. Figure 8B shows that pAkt (Ser-473) content was significantly reduced in the aorta of diabetic rats compared with the control groups for both sexes, whereas total Akt protein content remained unaffected by diabetes status, regardless of sex (Figure 8A).

**FIGURE 9:**
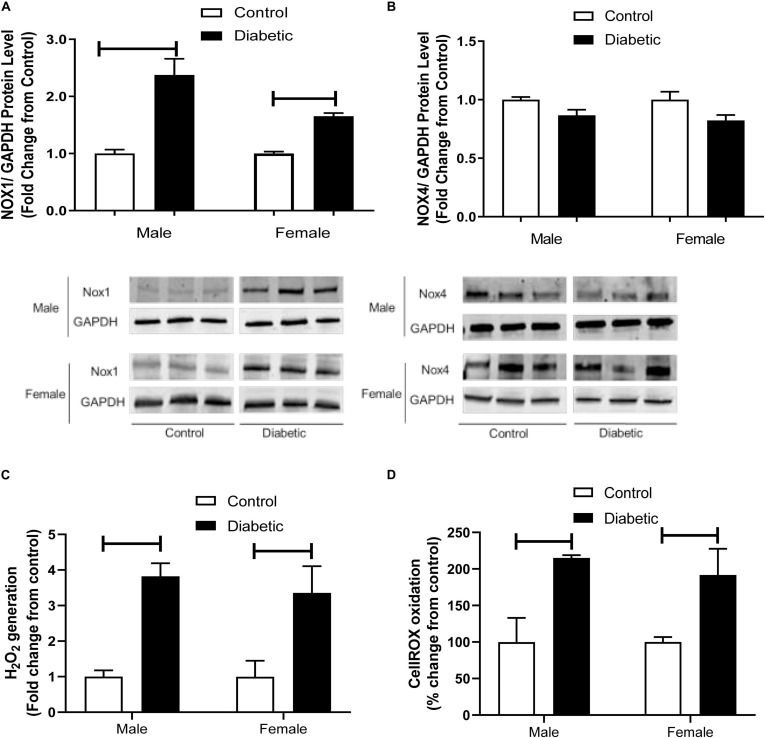
Western blot analysis of NOX1 and NOX4 expression in intact aorta, and oxidant generation in primary aortic endothelial cells isolated from control and UCD-T2DM rats. Protein levels of aortic NADPH oxidases (NOX1) **(A)** and NOX4 **(B)** from the samples of male and female control and diabetic rats. NOX1 and NOX4 were quantified by densitometry and normalized to corresponding GAPDH. To show representative bands, images from different parts of the same gel have been juxtaposed, indicated by white dividing lines. Hydrogen peroxide (H_2_0_2_) **(C)** and intracellular oxidant (CellROX oxidation) **(D)** generation in primary aortic endothelial cells isolated from male and female control and diabetic rats. Values are presented as mean ± SEM. Each bar represents the values obtained from *n* = 4–5 animals per group for NOX expression and *n* = 3–4 animals per group for oxidant generation studies. Capped lines indicate significant differences between two groups (*p* < 0.05), analyzed by unpaired Student’s *t*-test.

To further investigate the possible mechanisms underlying the elevated responses to contractile agents in this model, the protein expression of NADPH oxidase (NOX) subtypes NOX1 and NOX4 was measured. NOX1 expression was significantly elevated in aortic tissues from diabetic groups, regardless of sex (1.5-fold in male diabetic and 1-fold in female diabetic rats, Figure 9A). However, NOX4 expression showed no significant differences among all experimental groups (Figure 9B).

Lastly, to examine whether the elevated expression of NOX1 in aortic tissues of diabetic rats was associated with elevated basal ROS levels in this group, intracellular and extracellular ROS generation was measured in primary endothelial cells isolated from aortic tissues using Amplex Red and CellROX assays. Both assays demonstrated that ROS generation was higher in the endothelial cells isolated from arteries of diabetic groups compared with those in the non-diabetic controls, regardless of sex (Figures 9C,D).

## Discussion

The present study demonstrates that aortic function in UCD-T2DM rats is altered in both sexes. It also provides the first evidence of sexual dimorphism in aortic relaxation in UCD-T2DM rats.

In the current study, while both male and female diabetic rats had higher body weight and hyperglycemia compared with non-diabetic control rats, the female diabetic group exhibited higher adiposity, triglyceride, and insulin levels than control or male diabetic rats. This is consistent with the results from our previous study (Shaligram et al., 2020). Similarly, Ohta et al. (2014) which reported elevated blood insulin levels in spontaneously diabetic torii (SDT) female rats compared with SDT male rats. In the current study, the ISI was lowered in diabetic groups, irrespective of sex. However, when compared to male diabetic rats, female diabetic rats exhibited a lower ISI. Accordingly, here, we showed that insulin signaling was impaired in the aortic tissues in diabetic groups in both sexes. Notably, aortic IRS-1 was reduced to a similar extent in both diabetic groups, but IRS-2 was reduced only in the female diabetic group. It has been reported that the downregulation of IRS-2 levels in endothelial cells is induced by hyperinsulinemia in obese subjects (Kubota et al., 2011). Similarly, our results on elevated insulin levels in female diabetic rats may suggest that the decreased IRS-2 expression observed in the aorta could be in part due to hyperinsulinemia in this group.

In T2D, impaired (Sakamoto et al., 1998), enhanced (Zhong et al., 2012), or preserved (Bohlen and Lash, 1995) EDV has been reported. Here, a slight but significant decrease in maximum relaxation to ACh was observed in aortic rings from female UCD-T2DM rats compared to their respective controls. However, an intriguing observation of this study was that aortic rings from male diabetic animals exhibited a potentiation in EDV compared with that in male controls. Similar observations were also made by our group using Zucker diabetic fatty (ZDF) male rats. Specifically, obesity-induced diabetes (ZDF model) significantly impaired relaxation responses to ACh in aortic rings taken from females, but potentiated the relaxation in males (data not shown). In accordance with our current study, Zhong et al. (2012) reported elevated relaxation responses to ACh in aortic rings of GK male rats. On the other hand, Kazuyama et al. (2009) and Nemoto et al. (2011) reported an impaired EDV in aortic rings from GK male rats.

It has been well established that in conduit arteries, NO plays a major role in EDV (Shimokawa et al., 1996; Gao et al., 2011). The impaired EDV may result from either a decreased NO production or an increased inactivation of NO by ROS. It has been reported that T2D reduces the synthesis of NO in rat aorta by phosphorylation of eNOS at Ser-1177 (Nemoto et al., 2011). Here, we did not directly measure NO production, but our data show that the expression of the active, phosphorylated form of eNOS is decreased while the expression of NOX1 and ROS generation are increased in aortas from diabetic groups in both sexes, suggesting that decreased NO bioavailability may in part contribute to reduced responses to ACh in diabetic female arteries. However, elevated ACh responses in diabetic male aorta cannot be attributed to decreased NO due to decreased eNOS activation or elevation of ROS, suggesting that other factors may be involved.

There is an established negative regulatory effect of NO on EDHF synthesis (Bauersachs et al., 1996; Brandes et al., 2000), and an augmented EDHF response was also shown to compensate for the loss of NO in arteries in diabetic rats (Garland et al., 1995; Malakul et al., 2008). In agreement with those studies that demonstrate compensatory interactions between pathways, the potentiation of the ACh response (regardless of decreased eNOS activity) in aortic rings from the male diabetic group suggests that other vasodilatory molecules besides NO may be involved in ACh relaxation in this group.

In the present study, we showed that the inhibition of COX metabolites by Indo did not alter relaxation responses to ACh significantly in aortic rings of any of the four experimental groups. Consistent with these results, Malakul et al. (2008) reported that hypercholesterolemia and type 1 diabetes did not have any effect on COX-mediated EDV in rat aortas. Here, addition of ODQ completely abolished the EDV in aortic rings of control groups of both sexes as well as female, but not male, diabetic groups, suggesting that EDV is solely mediated by NO acting on the sGC (NO-dependent cGMP) pathway in the above-mentioned groups. Although the vasodilatory effect of NO on vascular smooth muscle is mainly mediated by cGMP (*via* a cGMP-dependent K^+^-channel activation) (Taylor et al., 2001), a direct effect of NO on Ca^2+^-dependent K^+^-channels (Bolotina et al., 1994) and L-type calcium current (Summers et al., 1999) without requiring cGMP has also been demonstrated. Here, the slight remaining Indo-ODQ-resistant relaxation in the aorta of male diabetic rats was unaffected by L-NNA, suggesting that NO-independent cGMP does not play a role in EDV in this group. An alternative explanation for the relaxation resistance to sGC and NOS inhibition in male diabetic aortas may be the contribution of other factors (NO- and PGI_2_-independent) on relaxation in this group. Similarly, Malakul et al. (2008) reported a potential role of EDHF in EDV in aortas of streptozotocin-induced type 1 diabetic male rats. However, they did not include females in their studies to determine whether there was a sex effect in the type 1 diabetic rat aorta. There are also other reports of a decreased NO-dependent relaxation response and increased EDHF activity in saphenous arteries (Chadha et al., 2010) and carotid arteries (Leo et al., 2010) of high-fat diet-induced obese and type 1 diabetic male rats, respectively.

Epidemiological studies suggest that males are at higher risk for CVD compared to age-matched females during their reproductive years (Liu et al., 2003). This sex difference has been attributed to estrogen’s protective effect in females and/or a detrimental androgen effect in males (Thompson and Khalil, 2003). However, a growing body of evidence suggests that androgens exhibit protective actions on the cardiovascular system (Nettleship et al., 2009). Administration of testosterone has been shown to induce both endothelium-dependent and independent vasorelaxation in rabbit aorta (Yue et al., 1995), rat aorta (Costarella et al., 1996), and porcine coronary artery (Crews and Khalil, 1999).

Here, our data show that control female aortas exhibit greater ACh-mediated vasorelaxation compared to male aortas. This is in accordance with our previous report on the sex difference in rat aortic relaxation (Rahimian et al., 1997); however, we have now extended these findings in reporting that under the T2D condition, beneficial effects of female hormones could be lost, yet, intriguingly, male aortas exhibit greater ACh-mediated relaxation.

The K_Ca_ currents are mainly mediated by IK_Ca_ and SK_Ca_ channels (Brähler et al., 2009) in conduit and resistance-sized arteries in many species, including humans (Taylor et al., 2001; Félétou, 2009; Grgic et al., 2009). Taylor et al. (2001) reported that NO-independent relaxations evoked by ACh in rabbit conduit arteries were sensitive to a combination of SK_Ca_ and IK_Ca_ channel blockers. Although the effects of SK_Ca_ and IK_Ca_ channel blockers were not examined in the current study design, our data on the significant increase in expression of IK_Ca_ and SK_Ca_ channels in male diabetic arteries suggest that the slight NO- and PGI_2_-independent relaxation observed in this group may be associated with these channels. In a preliminary functional study, further examination of IK_Ca_ and SK_Ca_, using selective inhibitors of these channels, suggested a role for IK_Ca_ in ACh-induced relaxation in aorta of male diabetic rats (data not shown). These results are also in accordance with Schach et al. (2014) who reported an elevation of expression and contribution of IK_Ca_ in mesenteric arteries of male ZDF rats. In the current study, we observed no significant differences in the expression of IK_Ca_ channels in aorta from female diabetic compared with female control animals (*n* = 5–6, data not shown). It is also important to note that in aorta from males, the contribution of a NO-independent factor to the ACh response was only observed in the diabetic state and not in the control (or healthy) state. Sandow et al. (2009) reported that EDHF is present in aortas of juvenile rats (Martínez-Orgado et al., 1998) and disease models (such as hypercholesterolemic, diabetic, hypertensive, and with altered estrogen levels), but absent in healthy adult aorta (Kagota et al., 2000; Matsumoto et al., 2004; Woodman and Boujaoude, 2004; Malakul et al., 2008).

Besides the possibility of a modified contribution of NO, alteration of EDV to ACh in aortas of the UCD-T2DM model could be explained by changes in smooth muscle responsiveness to NO or contractile agents. However, our data showed that SNP (a NO donor)-induced relaxation of endothelium-denuded aortic rings was not altered in male or female diabetic groups. Similarly, Nemoto et al. (2011) observed no significant difference in SNP-induced relaxation in aortic rings of male GK rats. This suggests that smooth muscle responsiveness to NO in the aorta was not affected in UCD-T2DM rats. On the other hand, in the current study, the sensitivity and maximum tension to PE were enhanced significantly in aortic rings of UCD-T2DM groups compared with their respective controls, regardless of sex (Figure 5). The elevated PE response may in part explain the slight but significant decrease in the maximum relaxation in ACh responses in aorta of female diabetic rats. However, it is important to note that regardless of increased PE-induced contraction, the ACh response was enhanced in male diabetic arteries. This, therefore, excludes the diminished PE contractile responsiveness as the cause of the increased ACh responses observed in male diabetic arteries. Nevertheless, our data on PE responses are in line with previous findings that type 1 diabetes results in increased vascular contraction in rat aortas (Abebe et al., 1990) and mesenteric arteries (White and Carrier, 1990).

Phenylephrine may indirectly stimulate endothelial cells to release NO *via* a signal transmitted either through myoendothelial gap junctions (Dora et al., 2000; Jackson et al., 2008) or by mechanical stress (Fleming et al., 1999). Therefore, the elevated contractile responses to PE observed in UCD-T2DM male and female rats may in part result from a decreased release of NO from the endothelium during smooth muscle contraction or an enhanced release of contracting factors (Zhang et al., 2012).

In the current study, we assessed the role of endothelium-derived NO by measuring the difference in the degree of PE-induced contraction in the absence and presence of L-NAME (Hayashi et al., 1992; Han et al., 2016). Pretreatment with L-NAME caused a significantly lower potentiation of the PE response in aortic rings from UCD-T2DM rats, regardless of sex (Figures 6B,D) compared with their controls. This suggests that decreased basal NO activity may in part be responsible for the elevated PE contractile responsiveness in UCD-T2DM rats in both sexes. Here, our study was limited in that we did not directly measure basal NO level. Nevertheless, consistent with an important role for eNOS phosphorylation on serine 1177 by Akt in regulating basal NO release (Scotland et al., 2002; Kobayashi et al., 2004), a reduction in eNOS expression by phosphorylation at Ser-1177 was observed in aortas from diabetic rats in both sexes compared with their controls. Additional studies will be needed to document the direction and magnitude of these interactions along with the relative importance of NO to elevated contractile responses in UCD-T2DM male and female rats.

Insulin resistance is a key element in the pathogenesis of T2D (Ormazabal et al., 2018). Insulin resistance is associated with endothelial dysfunction by several mechanisms including increased production of pro-inflammatory vasoconstrictor factors and oxidative stress (Schneider et al., 2000; Del Turco et al., 2011). Previous studies on experimental models of insulin resistance revealed impaired insulin-mediated PI3K/Akt-dependent signaling in the vasculature (Jiang et al., 1999). Our data on the significant decrease in expression of IRS and pAkt (a downstream mediator of IRS and upstream of eNOS phosphorylation at Ser-1177) in aortic tissues of diabetic animals in both sexes, suggest that the decreased peNOS levels in the diabetic aorta could arise from altered activation by pAkt.

Finally, it has also been reported that in diabetes, oxidative stress and superoxide radical production derived from insulin resistance may play a crucial role in enhancing the contracting responses (Shi et al., 2007). Superoxide scavenges NO, decreasing its bioavailability (Rubanyi et al., 1986), and elevating endothelium-dependent contractions. In the present study, we determined expression of NOX proteins, a source of superoxide. Vascular walls express high levels of NOX1, NOX2, and NOX4 (Griendling et al., 2000). NOX1 is mainly expressed in large conduit vessels (Lassègue et al., 2001), whereas NOX2 is more highly expressed in resistance vessels (Touyz et al., 2002). Here, we observed an elevated expression of NOX1 in aorta from diabetic groups, irrespective of sex, whereas NOX4 expression was not changed. Youn et al. (2012) reported that the activation of NOX1 was associated with eNOS uncoupling and endothelial dysfunction in streptozotocin-induced type 1 diabetic mice aorta. Furthermore, Gray et al. (2013) reported that genetic deletion of NOX1 in diabetic mice led to reduced diabetes mellitus symptoms, suggesting a key role of NOX1-derived ROS in diabetes. Consistent with these results is the observation that ROS generation in aortic primary endothelial cells isolated from diabetic rats was higher than in cells isolated from control animals, regardless of sex. Taken together, our results on NOX1 upregulation and increased ROS generation in diabetic arteries suggest that the elevation of responses to PE observed in diabetic animals in both sexes may be partially due to the reduced NO bioavailability or increased in generation of potential vasoconstrictor substances (such as superoxide anions).

## Conclusion

This study represents the first report showing that the aortic function in UCD-T2DM rats is altered in both sexes. Our data suggest that decreased insulin sensitivity, possibly *via* pAkt-dependent signaling and enhanced oxidative stress, may contribute to the elevated contractile responses in aorta of this model of T2D, regardless of sex. We also showed sex differences in aortic relaxation in this model. Specifically, our data show that under the T2D condition, beneficial effects of female hormones could be lost, yet, intriguingly, male aortas exhibit greater ACh-mediated relaxation. Additional studies will be needed to identify an underlying mechanism for the sex-specific differences observed in the aortic relaxation in this model.

**FIGURE 10:**
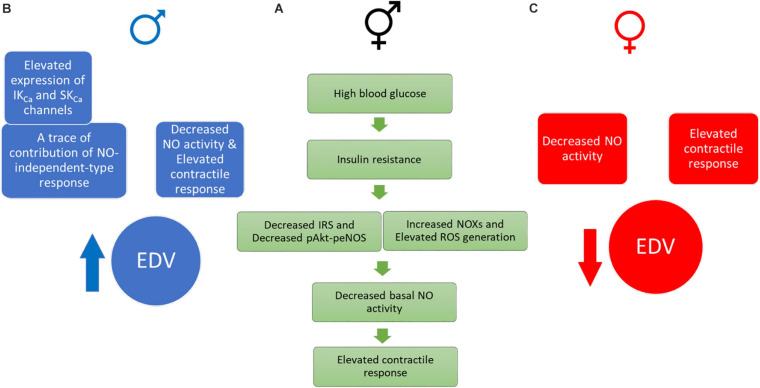
Proposed drivers of an elevated contractile response in UCD-T2DM rat aortas. (**A** green one) Impaired insulin signaling, elevated ROS generation, and decreased basal NO activity may drive elevated contractile responses in aortic rings of both male and female UCD-T2DM rats. (**B** blue one) Male UCD-T2DM rat aortas display enhanced EDV (despite elevated contractile responses) along with elevated IK_C__a_ and SK_C__a_ channel expression and traces of NO-independent responses. (**C** red one) Female UCD-T2DM rat aortas display impaired EDV, possibly due to decreased NO activity and enhanced contractile responses. IRS, insulin receptor substrate; pAkt, phosphorylated V-Akt murine thymoma viral oncogene homolog-2; peNOS, phosphorylated endothelial nitric oxide synthase; NOX, NADPH oxidase; ROS, reactive oxygen species; NO, nitric oxide; IKCa, intermediate-conductance calcium- activated potassium channel; SKca, small-conductance calcium-activated potassium channel; EDV, endothelium-dependent vasorelaxation.

Figure 10 depicts our proposed scheme based on the data presented in this report. Briefly, the elevation of contractile responses in aortic rings of both male and female UCD-T2DM could result from decreased basal NO activity, possibly due to the impaired insulin-mediated pAkt-peNOS dependent signaling and/or increased oxidative stress in this model (A). In the meantime, aortic vasorelaxation was elevated in aortic rings from male UCD-T2DM rats (B), but slightly impaired in female UCD-T2DM rats (C). The elevated vasorelaxation response in aortic rings from male diabetic rats (despite elevated contractile responses in this group) was accompanied by the elevated IK_Ca_ and SK_Ca_ channel expression and trace a of NO-independent responses (B). However, the decreased relaxation in female diabetic aortas could be in part attributed to the decreased NO activity and elevated contractile responses in this group (C).

## Data Availability Statement

The original contributions presented in the study are included in the article/supplementary material, further inquiries can be directed to the corresponding author/s.

## Ethics Statement

The animal study was reviewed and approved by the University of the Pacific and the University of California, Davis Institutional Animal Care and Use Committee.

## Author Contributions

FA designed and performed the majority of the experiments and drafted the manuscript. MR and SS assisted with the animal experiments. PH, KS, and JG generated the UCD-T2DM rat models and provided the intellectual input and critical reading of the manuscript. JV-M and KA performed the ROS experiments and provided the intellectual input. KA was also involved in editing the manuscript. RR was involved with the conception and design of research, revising the manuscript, and approving the final draft of manuscript. All the authors contributed to the article and approved the submitted version.

## Conflict of Interest

The authors declare that the research was conducted in the absence of any commercial or financial relationships that could be construed as a potential conflict of interest.
